# *In situ* electrochemical observation of anisotropic lattice contraction of La_0.6_Sr_0.4_FeO_3−*δ*_ electrodes during pulsed laser deposition†

**DOI:** 10.1039/d2cp04977e

**Published:** 2022-12-01

**Authors:** Christoph Riedl, Matthäus Siebenhofer, Sergej Ražnjević, Andreas Ewald Bumberger, Zaoli Zhang, Andreas Limbeck, Alexander Karl Opitz, Markus Kubicek, Jürgen Fleig

**Affiliations:** Institute of Chemical Technologies and Analytics, TU Wien Vienna Austria matthaeus.siebenhofer@tuwien.ac.at; Centre for Electrochemistry and Surface Technology, CEST, Wr Neustadt Austria; Erich Schmid Institute for Materials Science Leoben Austria

## Abstract

La_0.6_Sr_0.4_FeO_3−*δ*_ (LSF) electrodes were grown on different electrolyte substrates by pulsed laser deposition (PLD) and their oxygen exchange reaction (OER) resistance was tracked in real-time by *in situ* PLD impedance spectroscopy (i-PLD) inside the PLD chamber. This enables measurements on pristine surfaces free from any contaminations and the direct observation of thickness dependent properties. As substrates, yttria-stabilized zirconia single crystals (YSZ) were used for polycrystalline LSF growth and La_0.95_Sr_0.05_Ga_0.95_Mg_0.05_O_3−*δ*_ (LSGM) single crystals or YSZ single crystals with a 5 nm buffer-layer of Gd_0.2_Ce_0.8_O_2−*δ*_ for epitaxial LSF film growth. While polycrystalline LSF electrodes show a constant OER resistance in a broad thickness range, epitaxially grown LSF electrodes exhibit a continuous and strong increase of the OER resistance with film thickness until ≈60 nm. In addition, the activation energy of the OER resistance increases by 0.23 eV compared to polycrystalline LSF. High resolution transmission electron microscopy (HRTEM) and X-ray diffraction (XRD) measurements reveal an increasing contraction of the out-of-plane lattice parameter in the epitaxial LSF electrodes over electrode thickness. Defect thermodynamic simulations suggest that the decrease of the LSF unit cell volume is accompanied by a lowering of the oxygen vacancy concentration, explaining both the resistive increase and the increased activation energy.

## Abstract 2

The electrochemical effects of anisotropic lattice deformation during La_0.6_Sr_0.4_FeO_3−*δ*_ growth by pulsed laser deposition are investigated by *in situ* impedance spectroscopy.

## Graphical abstract

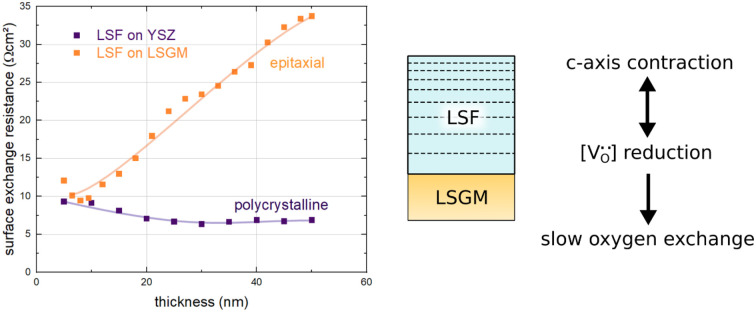

## Introduction

Lowering the operation temperature of solid oxide fuel and electrolysis cells (SOFCs and SOECs) to intermediate temperatures (450–600 °C) is one of the main goals of current research activities in this field, as operation temperatures enable the usage of cheaper materials, reduce start-up times and improve the long-term stability of the cells.^1,2^ However, virtually all important electrochemical processes in the cells are thermally activated and thus a lower operation temperature inevitably leads to higher resistive losses, limiting the current output and efficiency of the devices.^3^ Thus, improved or novel cathode materials are of high importance for a broader application of intermediate temperature solid oxide cells (SOCs).

While currently mostly La_1−*x*_Sr_*x*_MnO_3−*δ*_-based or La_1−*x*_Sr_*x*_Co_*y*_Fe_1−*y*_O_3−*δ*_-based cathode materials are applied in SOFCs,^4^ many studies have also investigated numerous other mixed ionic and electronic conducting (MIEC) electrodes, such as La_1−*x*_Sr_*x*_CoO_3−*δ*_ (LSC),^5–7^ La_1−*x*_Sr_*x*_FeO_3−*δ*_ (LSF),^8,9^ SrTi_*x*_Fe_1−*x*_O_3−*δ*_ (STF),^10–13^ Pr_*x*_Ce_1−*x*_O_2−*δ*_ (PCO),^14–16^ Nd_2_NiO_4+*δ*_ (NNO)^17–19^ or Sm_1−*x*_Sr_*x*_CoO_3−*δ*_ (SSC)^20^ in order to test their applicability as SOFC cathodes or SOEC anodes. These materials are promising candidates as electrode materials, but also suffer from disadvantages, such as slow overall oxygen exchange kinetics and/or strong degradation susceptibility. For instance, measurements on dense LSC thin film electrodes revealed very low OER resistances of the surface exchange reaction (<1 Ω cm^2^ at 600 °C in air) in their pristine state, but unfortunately also a continuous and severe increase of the OER resistance over time.^21^

A major goal of current research activities on thin film electrodes is an improved mechanistic understanding of the OER on electrode materials and knowledge driven material design of porous, real-life electrodes may greatly benefit from such fundamental investigations. Recent studies found the surface concentration of different point defects (*e.g.*: electrons, holes, oxygen vacancies) to strongly influence the oxygen exchange rate.^16,22–24^ Thus, tuning defect concentrations of the electrode surface (*e.g.* by surface decorations) will significantly alter the oxygen exchange activity.^6,23,25^ In general, the surface chemistry seems to be a deciding factor for the overall oxygen exchange activity and a wide variety of other modifications such as thermal activation,^26^ DC polarization^27^ or liquid surface treatments^28^ have been used to optimize the surface for the oxygen exchange reaction.

Another approach to alter the oxygen exchange kinetics on oxide surfaces makes use of lattice strain. Impedance measurements on NNO^17^ and LSC^29^ electrodes have shown that tensile strain can accelerate the oxygen exchange activity, while for compressively strained electrodes decreased oxygen exchange kinetics were observed. In several thin film studies under tensile strain, not only faster oxygen exchange kinetics, but also higher concentrations of oxygen vacancies in the bulk of the electrode were found.^29–31^ Similarly, ^18^O isotope exchange measurements revealed an enhanced OER rate and a higher oxygen vacancy diffusion coefficient in LSC thin films under tensile strain compared to compressively strained LSC films.^32,33^ In addition, in a recent study, the effect of strain on the surface defect concentrations of LSF was investigated and it was demonstrated that surfaces under tensile strain tend to be considerably more reduced compared to the unstrained state.^34^ Moreover, HRTEM studies showed oxygen vacancy ordering in LSC films under tensile strain^33^ and DFT studies suggested that tensile biaxial strain reduces the oxygen vacancy migration barrier across the system,^35^ and lowers the oxygen vacancy formation enthalpy.^30^ The results from these experiments on strained thin films are in good accordance with recent mechanistic studies, which quantified how the oxygen vacancy concentration enters the rate law of the oxygen exchange reaction.^16,22^

In this study, epitaxial and polycrystalline LSF thin film electrodes were grown stepwise by pulsed laser deposition (PLD) and their OER resistance was measured by *in situ* impedance spectroscopy during pulsed laser deposition (i-PLD). This approach allows one to grow an electrode material and simultaneously perform impedance measurements. Thus, thickness dependent changes of the electrode properties can be tracked during growth, unveiling information which is usually hardly accessible by standard *ex situ* measurements. In addition, i-PLD measurements enable impedance measurements on pristine electrodes free from any external contaminations or electrode degradation, which gives further insights into the electrodes OER kinetics. Employing this technique, an unexpected increase of the OER resistance of epitaxial LSF thin films with increasing film thickness as well as an increased activation energy with regard to polycrystalline films are discovered. Additional high resolution transmission electron microscopy measurements (HRTEM) and X-ray diffraction (XRD) measurements clarify the evolution of the LSF lattice geometry during thin film growth and tie the observed decline of the oxygen exchange kinetics to a continuously decreasing unit cell volume in epitaxial thin films. Considering that a reduced unit cell volume can lead to an increased oxygen vacancy formation enthalpy, defect thermodynamic calculations are successfully employed to explain the observed increase of the OER resistance and the altered activation energy.

## Methodology

### Experimental methods

In the course of the experiments, three different substrates were used: (i) (100)-oriented yttria stabilized zirconia (YSZ, 9.5 mol% Y_2_O_3_, Crystec GmbH, Germany), (ii) (001)-oriented La_0.95_Sr_0.05_Ga_0.95_Mg_0.05_O_3−*δ*_ (LSGM) single crystals (both 5 × 5 × 0.5 mm^3^) grown by the Czochralski technique^36^ and (iii) YSZ single crystals from (i) with a 5 nm Gd_0.2_Ce_0.8_O_2−*δ*_ (GDC) buffer layer. The GDC buffer layer was deposited *via* pulsed laser deposition (PLD) at a substrate temperature of 600 °C in 0.01 mbar O_2_ at a substrate-target distance of 6.0 cm and a laser frequency of 1 Hz. For all i-PLD measurements, Ti/Pt grids (5 nm Ti, 100 nm Pt, 15/5 μm holes/mesh) were prepared on both sides of the substrates by lift-off photolithography and magnetron sputtering. The thin Ti layer acts as an adhesion aid between oxide and Pt. As a counter electrode, nanoporous La_0.6_Sr_0.4_CoO_3−*δ*_ was deposited on one of the Ti/Pt grids (bottom grid) *via* PLD at 450 °C in 0.4 mbar O_2_ at a substrate-target distance of 5.0 cm.^37,38^ During the actual i-PLD measurements, La_0.6_Sr_0.4_FeO_3−*δ*_ (LSF) was deposited onto the top Ti/Pt grid *via* PLD at a temperature of 600 °C, a pressure of 0.04 mbar O_2_, a substrate-target distance of 6.0 cm and a laser frequency of 2 Hz. For all depositions, a KrF Excimer Laser (*λ* = 248 nm, Lambda Physics, COMPex Pro 201) with a laser fluence of ∼0.9 J cm^−2^ was used.

Impedance spectroscopic measurements inside the PLD chamber were performed on a specially built heating stage^39^ with an Alpha-A High Performance Frequency Analyzer and Electrochemical Test Station POT/GAL 30V/2A setup (Novocontrol Technologies) in a frequency range from 10^6^ to 10^−1^ Hz. For a more detailed description of i-PLD measurements, the reader is referred to earlier studies employing this technique.^22,29^ The sample temperature was measured *via* the high frequency intercept of the recorded impedance spectra, consisting of the only very slightly temperature dependent wiring resistances of the setup and the Ti/Pt grid as well as the strongly temperature dependent electrolyte resistance.^40^

X-ray diffraction (XRD) measurements were performed with an Empyrean X-ray diffractometer (Malvern Panalytical). For all measurements, a hybrid monochromator was used on the incident beam side and a GaliPIX3D area detector was used in the diffracted beam path (both Malvern Panalytical). Diffractograms were analyzed with Panalytical Highscore.^41^ For XRD measurements, samples were deposited with the same parameters without current collecting grids, except for LSF on LSGM, where (due to limited single crystal supply), the i-PLD sample was used during XRD measurements.

Atomic force microscopy (AFM) measurements were performed in tapping mode with a Nanoscope V multimode setup (Bruker) on a scan range of 1 × 1 μm^2^, directly after i-PLD measurements on the same samples. The AFM results were evaluated with Gwyddion^44^ to gain information about the surface morphology and crystallinity of the deposited thin films.

The lamella for HRTEM investigations was prepared from the same LSF/LSGM sample which has been measured with i-PLD (*cf.*Fig. 5) by lift-out technique^45^ on a Thermo Fisher Scios 2 DualBeam FIB/SEM with a Ga-ion beam operating at 30 kV accelerating voltage. For final thinning and polishing, the voltage was successively reduced to 5 kV and 2 kV. HRTEM images were acquired using an image-side Cs-corrected JEOL 2100F field-emission transmission electron microscope operating at 200 kV. In order to evaluate lattice parameters, geometrical phase analysis (GPA) technique was used. Each measurement is an average of the area profile spanning about 28 unit cell distances in width (≈11 nm).

## Results

### Structural characterization and surface morphology

**Fig. 1:**
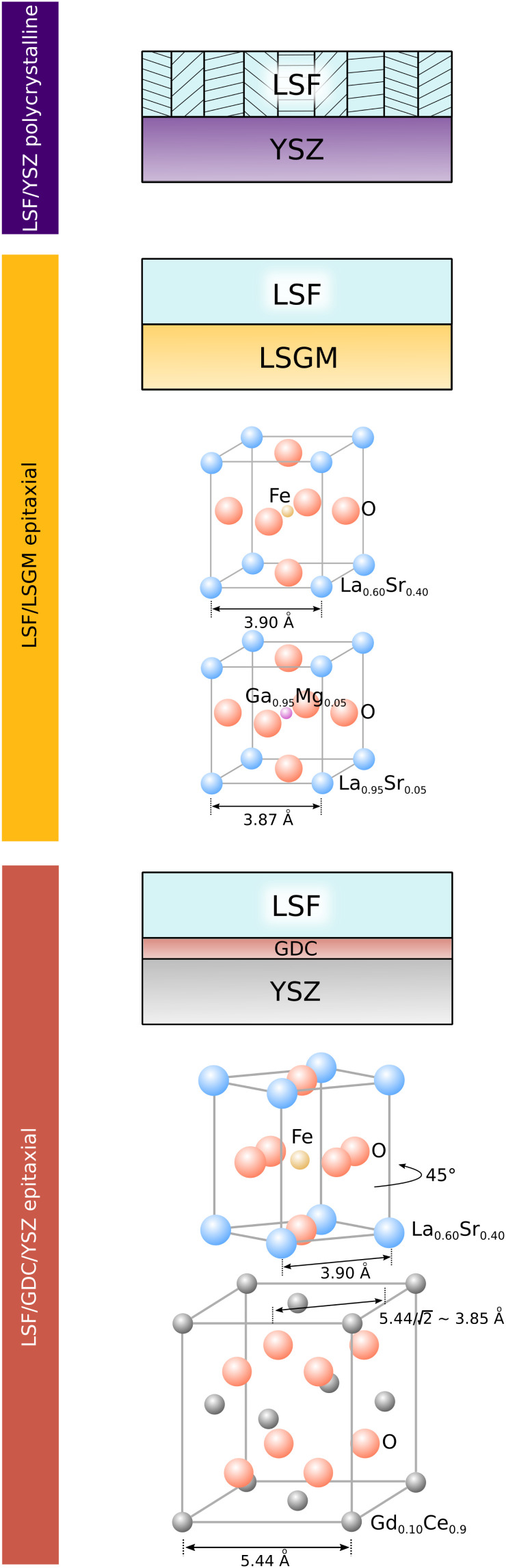
Sketch showing the structure of LSF electrodes grown on YSZ, LSGM and YSZ–GDC. Lattice parameters of substrates refer to measurements from literature.^36,42,43^

To correlate structural and electrochemical properties of LSF thin films, detailed information on the film structure is required. In this study, polycrystalline and epitaxial LSF thin films are investigated. Polycrystalline LSF thin films have been thoroughly investigated in literature^46,47^ and were grown on YSZ single crystals. In order to realize epitaxial film growth, we used LSGM and YSZ–GDC (YSZ single crystals with a 5 nm PLD grown buffer layer of Gd_0.2_Ce_0.8_O_2−*δ*_; cube-on-cube alignment) substrates, see sketch in Fig. 1. While for the perovskite based substrate LSGM (orthorombic, *Imma*), epitaxial growth of LSF is not surprising and was already shown in a previous study,^48^ it is not obvious that LSF can grow epitaxially on GDC. However, studies in literature have already shown the epitaxial growth of the structurally similar LSC on a GDC buffer layer^42,49,50^ with a 45° in-plane rotation. In our study, we could confirm that also LSF can grow epitaxially on YSZ–GDC.

**Fig. 2:**
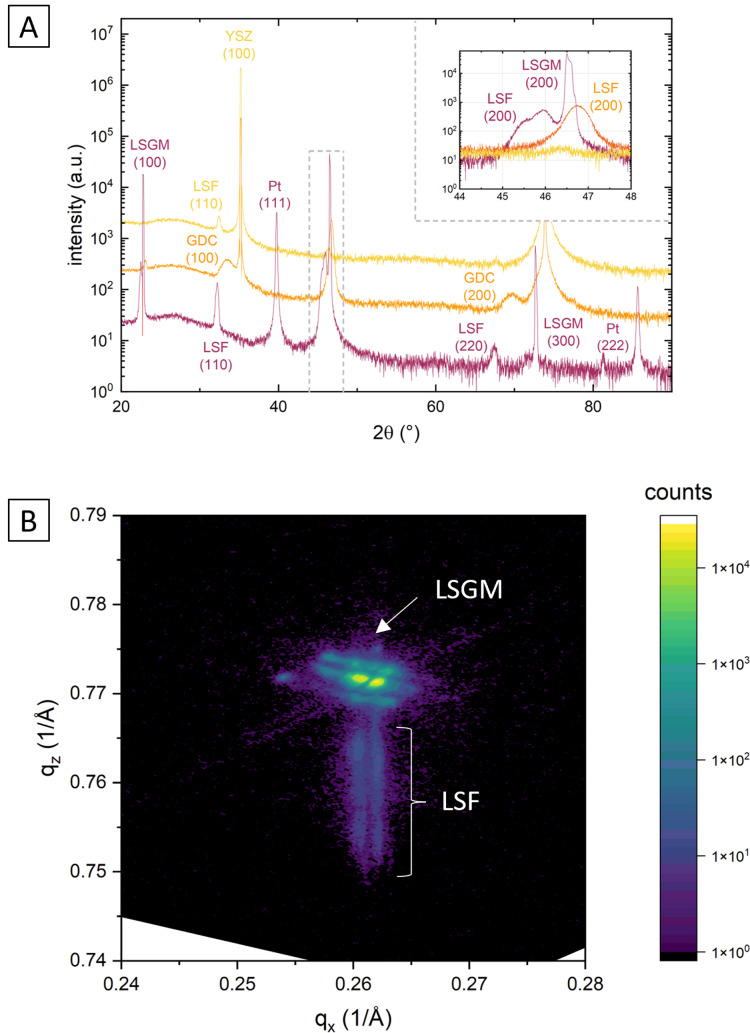
(A) *θ*–2*θ* scans of LSF on LSGM, YSZ–GDC and YSZ showing oriented growth on LSGM and GDC/YSZ and polycrystalline growth on YSZ; (B) reciprocal space map of an LSF thin film (50 nm) grown on LSGM.

This is demonstrated by X-ray diffraction in *θ*–2*θ* geometry and reciprocal space mapping, as well as atomic force microscopy (AFM). From the *θ*–2*θ* scans in Fig. 2(A) it is apparent, that LSF on both LSGM and YSZ–GDC grows highly oriented along the [001] direction. Moreover, on LSGM, the quasi-cubic *c*-axis lattice parameter is larger (3.947 Å) than for LSF grown on YSZ–GDC (3.883 Å). Also, both peaks are broadened, indicating a relatively small crystallite size or a varying lattice parameter. The additional reflexes for LSF grown on LSGM corresponding to LSF (110) and Pt (111) are due to a platinum grid and the LSF film on top, which couldn’t be avoided as an i-PLD sample was used for XRD measurements due to limited sample supply. In addition, a reciprocal space map was recorded on LSF thin films grown on LSGM as substrate. The results show that the LSGM single crystal exhibits multiple domains with slightly varying lattice parameters. Furthermore, the LSF film peak is clearly visible below the single crystal reflex, indicating laterally compressive strain of the film. As can be seen in Fig. 2(B), the film peak is not distinctly separated, but is smeared out over a relatively wide range of *c*-axis parameters, which confirms a change of the lattice parameter over the film thickness.

The surface of dense LSF thin films grown on LSGM, YSZ–GDC and YSZ was investigated by AFM, see images in Fig. 4. LSF thin films on both LSGM and YSZ–GDC exhibited very flat surfaces with a root-mean-square (RMS) roughness of 0.24 nm on LSGM and 0.25 nm RMS roughness on YSZ–GDC. Also, HRTEM measurements (see below) do not show signs of grain boundaries, confirming epitaxial film growth. The surface of LSF grown directly on YSZ, however, showed a strongly granular structure and a comparatively rough surface (5.78 nm RMS roughness). We thus conclude that LSF grows epitaxially on LSGM and YSZ–GDC, oriented in the [001] direction, and in a polycrystalline structure on YSZ single crystals.

### i-PLD growth of epitaxial and polycrystalline LSF

**Fig. 3:**
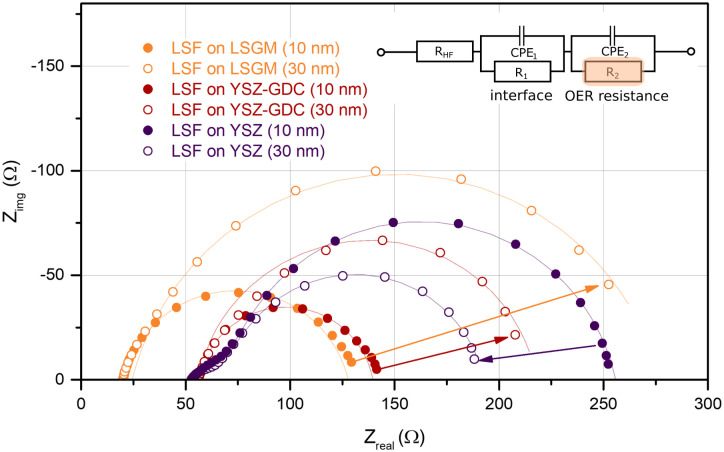
Impedance spectra (Nyquist plot) of LSF thin films after 2000 and 6000 pulses of LSF deposited on the different substrates LSGM, YSZ–GDC and YSZ, measured at 600 °C in 0.04 mbar O_2_. The inset displays the equivalent circuit used for fitting; CPE = constant phase element. *R*_1_‖CPE_1_ was only required for some of the spectra. The oxygen exchange reaction (OER) resistance *R*_2_ was analyzed in more detail.

In order to understand how the different crystallographic structures of LSF influence the oxygen exchange kinetics, the electrochemical properties of all three types of LSF (on LSGM, YSZ–GDC and YSZ) were tracked by impedance spectroscopic measurements during growth (i-PLD). In Fig. 3, exemplary impedance spectra are shown. All spectra exhibit a real-axis offset at high frequencies corresponding to ohmic resistances of the setup, the Ti/Pt grid and the electrolyte. The spectra in Fig. 3 correspond to a substrate temperature of 600 °C;^36,51^ different off-sets are due to different ionic conductivities of LSGM and YSZ single crystals. Furthermore, one dominant impedance feature was observed in all measurements, which corresponds to the oxygen exchange resistance at the surface of the growing working electrode in parallel to the chemical capacitance of the thin film.^13,17,22,52^ It is worth mentioning, that samples before LSF deposition also exhibit an impedance curve with one main feature corresponding to the oxygen exchange at the triple phase boundary of the current collecting grid. However, due to the much slower kinetics, the corresponding resistance is in the range of 10^4^ Ω. Upon growth of the LSF film, significant changes of this low frequency feature were found (see Fig. 5), which will be discussed in detail below. At the high frequency end of the main semicircle a slight shoulder was observed in some measurements, which did not change considerably with film thickness. In literature, similar shoulder-like features were attributed to interfacial resistances between the thin film and the substrate. This interpretation is also supported by the fact that the interfacial resistance was most prominent for LSF grown directly on YSZ, *i.e.* an interface which is prone to reaction.^53^

**Fig. 4:**
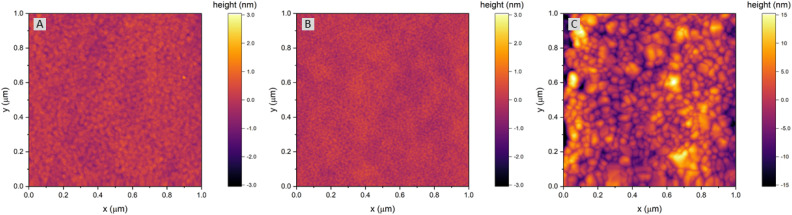
AFM images of LSF thin films grown on (A) LSGM, (B) YSZ–GDC and (C) YSZ. Please note the different height scale bars for LSF thin films grown on LSGM and YSZ–GDC *vs.* LSF thin films grown on YSZ.

Impedance spectra were repeatedly recorded after 1000 laser pulses, corresponding to ∼5 nm of deposited material. The area specific resistance of the oxygen exchange reaction (OER) was extracted from the main arc (*R*_2_) of the impedance spectra by using the equivalent circuit sketched in Fig. 3. We would like to emphasize that the resistance values found in this study by i-PLD measurements on polycrystalline LSF thin films are about an order of magnitude lower than values usually reported in literature during standard *ex situ* measurements, which is owed to the very clean measurement conditions inside the setup of the i-PLD. This low resistance is a common feature of all i-PLD investigations and was clarified in a recent study.^54^ Trace amounts of sulphur contaminants in the high ppb range are present in most measurement gases and, by fast adsorption on SOFC cathode surfaces, inhibit the oxygen exchange reaction in a variety of ways (site blockage, adsorption behaviour, surface potential change, defect concentration changes).

**Fig. 5:**
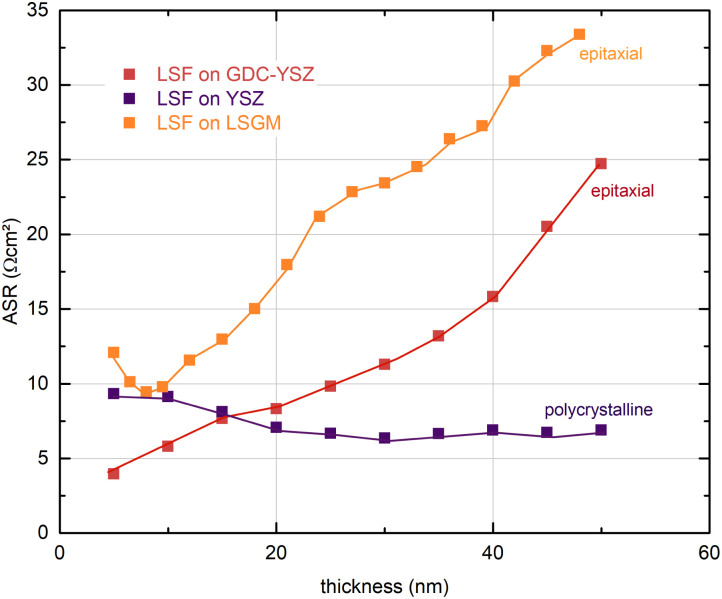
Thickness dependent area-specific oxygen exchange resistance (ASR) of dense LSF thin films on LSGM, YSZ/GDC and YSZ substrates measured by i-PLD, at 600 °C and 0.04 mbar oxygen partial pressure.

In Fig. 5, the thickness dependent area-specific oxygen exchange resistance (ASR from *R*_2_) is shown for LSF grown on different substrates. It is apparent that the oxygen exchange resistance develops rather differently for polycrystalline and epitaxial LSF thin films. On polycrystalline LSF, a slight variation in the beginning is followed by an almost constant OER resistance after around 20 nm. The OER resistances of the two epitaxial thin films grown on LSGM and YSZ–GDC, on the other hand, exhibit a strong increase over film thickness by a factor of about 3–6. This is surprising, as the overall composition of the thin film should not change with its thickness nor should degradation phenomena play a role, since every 5 nm the surface is completely renewed by PLD. This behaviour is also different from epitaxial LSC thin films grown on LSGM, where an earlier study showed a constant ASR after a film thickness of about 30 nm was reached.^29^

To investigate this process for thicker films, a 100 nm LSF thin film electrode was grown on YSZ–GDC to reveal whether a saturation of the ASR can be observed. Fig. S1 in the ESI† shows that the polarization resistance increases up to a film thickness of about 60 nm and then decreases again, thus excluding bulk transport as the limiting process. This was confirmed by a second experiment, where the high-resistance LSF surface after 50 nm was covered with LSC and the resistance immediately decreased to a value typical for pure LSC. The combination of these results strongly indicates that the altered kinetics are caused by subtle differences in the LSF surface at different film growth stages.

**Fig. 6:**
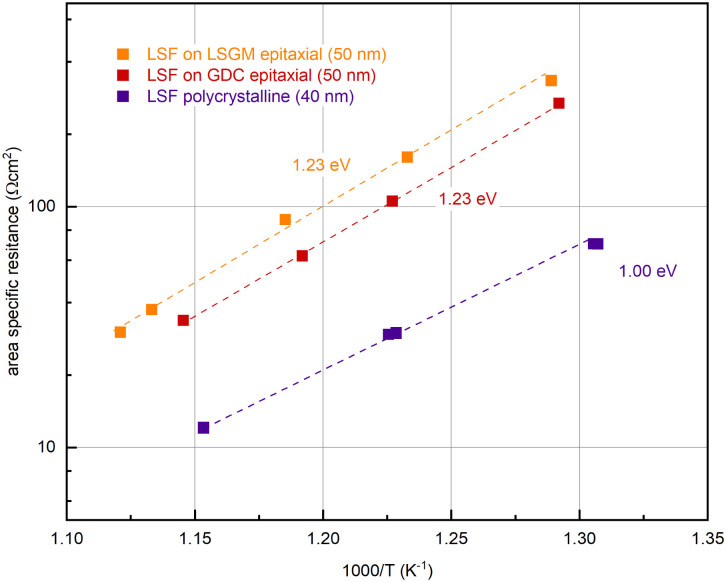
Determination of the activation energy at an oxygen partial pressure of 0.04 mbar of epitaxial LSF (grown on LSGM (orange) and GDC (red)) and polycrystalline LSF (purple).

In further experiments, the oxygen partial pressure and temperature dependence of the OER resistance (*R*_2_) of epitaxial LSF at 600 °C was compared with LSF grown on YSZ. While polycrystalline and epitaxially grown LSF show a very similar *p*O_2_ dependence of the OER resistance (see Fig. S2 in the ESI†), the activation energy differs between the two growth modes (see Fig. 6). On polycrystalline LSF, an activation energy of 1.00 eV was found, on LSF grown epitaxially on LSGM and YSZ–GDC the measured activation energy was considerably increased at 1.23 eV. As shown in ref. 22, the activation energy includes temperature dependences of defect concentrations as well as kinetic barriers. Different activation energies thus do not necessarily indicate a different reaction mechanism (which is also supported by the similar *p*O_2_ dependence). Rather, they can act as a sensitive descriptor of changes in the electrode defect chemistry. The different values found here may thus indicate a shift of the defect thermodynamics. A detailed discussion of the relationship between film thickness, growth mode, point defect chemistry and ASR will be given in the Discussion section below.

### High resolution transmission electron microscopy (HRTEM)

As i-PLD measurements revealed a substantial increase of the OER resistance with film thickness on epitaxial electrodes, HRTEM measurements were conducted to further investigate the structure of the electrode on an atomic level. In Fig. 8, HRTEM images of an LSF thin film grown on LSGM are shown. The interface between LSGM and LSF is clearly visible and HRTEM images confirm that the LSF thin film grows epitaxially and without grain boundaries on the single crystalline LSGM substrate. Moreover, no apparent dislocations were observed in the LSF thin film. It is worth mentioning that local fast Fourier transformation (FFT) found minor amounts of brownmillerite phase to be present in the thin film, however, it was equally distributed throughout the thin film and no correlation with film thickness was found. Thus, we assume that brownmillerite type phases do not play a decisive role in explaining our measurement results. (FFT patterns can be found in the ESI,† Fig. S3).

In addition, HRTEM measurements also enable the investigation of the LSF lattice parameters at different positions of the thin film. In order to gain statistically meaningful data, the lattice parameters were measured at 15 different lateral positions on the TEM lamella in close proximity to the LSGM interface (5 nm) and 35 nm away from the interface. These measurements reveal that the in-plane and out-of-plane lattice parameter evolve differently with the film thickness (see Fig. 8). The out-of-plane lattice parameter is significantly larger than the in-plane lattice parameter, confirming the laterally compressive strain imposed by the LSGM substrate. For the in-plane lattice parameter, HRTEM measurements found a value of 3.871 ± 0.006 Å close to the LSGM interface, which hardly changed with film thickness (3.873 ± 0.008 Å at 35 nm). For the out-of-plane lattice parameter, however, a significant change with film thickness was observed. The lattice parameter decreased from 3.973 ± 0.007 Å close to the interface to 3.946 ± 0.005 Å 35 nm away from the substrate, which corresponds to a decrease of the out-of-plane lattice parameter by about −0.68%. Considering that small amounts of strain have shown to alter the oxygen exchange kinetics considerably,^29,32,55^ we believe that this anisotropic lattice deformation could have a strong effect on the surface oxygen exchange kinetics (see below).

**Fig. 7:**
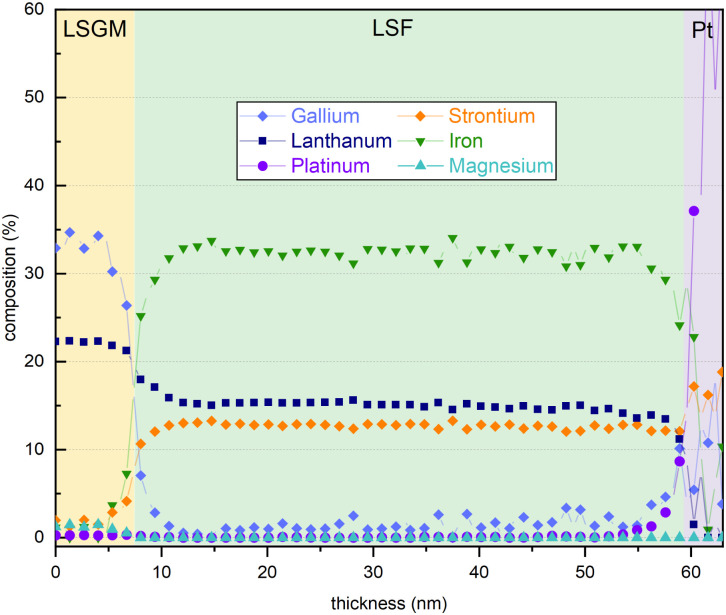
EDX line scan of an LSF thin film grown on a LSGM substrate with a Pt protection layer.

In addition, an energy dispersive X-ray (EDX) line profile was acquired to exclude that severe changes of the electrode composition over film thickness influence the electrode OER resistance (see Fig. 7). The interfaces between LSGM|LSF and LSF|Pt protection layer are clearly visible in the EDX line profile images. The platinum protection layer was applied on the electrode surface after electrochemical measurements to prevent damages during the FIB-cut. The interface between LSGM and LSF is not completely sharp which may indicate slight intermixing with the substrate, however, this could also be due to measurement inaccuracies during TEM investigations. In conclusion, the EDX analysis reveals that no clear compositional trend is observed in the cation stoichiometry across the film thickness. We also evaluated cation/oxygen ratios but, similarly, they do not show any significant trend with film thickness, rendering a strong change of cation vacancy concentrations unlikely. Thus, we cannot exclude minor changes of cation concentrations in the film but did not find any experimental evidence for significant compositional changes of the electrode which could explain the observed resistance change.

**Fig. 8:**
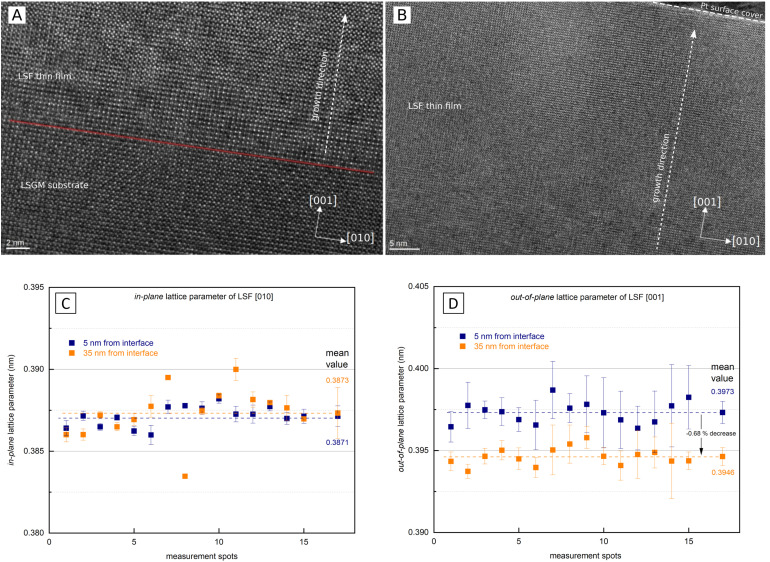
(A) HRTEM image of the interface region between LSGM and LSF (B) HRTEM image of the LSF thin film (surface near region visible). (C) Lattice parameter in the in-plane [010] direction 5 nm from the LSGM interface (blue) and 35 nm from the LSGM interface (orange). (D) Lattice parameter in the out-of-plane [001] direction 5 nm from the LSGM interface (blue) and 35 nm from the LSGM interface (orange).

### Comparison of lattice parameters and volume on polycrystalline and epitaxial LSF

**Fig. 9:**
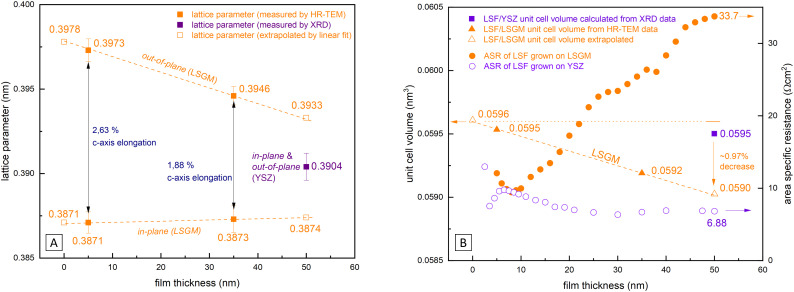
(A) Comparison of the in-plane and out-of-plane lattice parameters of LSF grown on LSGM measured by HRTEM with the lattice parameter of LSF grown on YSZ measured by XRD are shown. (B) Comparison of the lattice volume of polycrystalline *vs.* epitaxial grown LSF and the measured ASR.

In Fig. 9(A) the lattice parameters of epitaxial LSF (derived by HRTEM measurements, see Fig. 8) are compared with the pseudocubic lattice parameter of polycrystalline LSF (obtained by *θ*–2*θ* XRD measurements with the YSZ single crystal lattice parameter used as reference). Based on our HRTEM measurement results, the lattice parameters of epitaxial LSF were approximated assuming a linear relationship to extrapolate the lattice parameter decrease for the entire LSF thin film thickness (*i.e.* 0–50 nm; see dashed orange lines in Fig. 9(A)). For polycrystalline LSF grown on YSZ the lattice parameter is presumed to be constant over the entire electrode thickness (this assumption is supported by grazing incidence XRD measurements of polycrystalline LSF which do not show any peak broadening or other indications of a varying lattice parameter, see Fig. S4 in the ESI†).

From our approximation of the out-of-plane lattice parameter made in Fig. 9(A), the volume of the tetragonal LSF unit cell was calculated (see Fig. 9(B)) and a substantial decrease of the unit cell volume from 0 to 50 nm film thickness by ≈1% is found. As the out-of-plane lattice parameter and thus the LSF unit cell volume is the only property for which a significant dependence on the film thickness was measured, we suspect that the variation of this parameter is tied to the increase of the OER resistance on epitaxial LSF thin films during i-PLD measurements. In the following, we discuss possible effects which may result from this lattice deformation and which might affect the oxygen exchange kinetics.

## Discussion

A defect thermodynamic explanation must consider the following experimental results regarding the thickness dependent electrochemical properties of epitaxial LSF thin film electrodes upon i-PLD growth:

(i) A continuous increase of the OER resistance is observed for epitaxial LSF thin films grown on LSGM or YSZ–GDC substrates, while little variation of the OER resistance is found for polycrystalline LSF electrodes grown on YSZ. At a film thickness of 50 nm the OER resistance of epitaxial LSF (grown on LSGM) is about a factor of 6 higher than that of polycrystalline LSF, (see Fig. 5). Experimental results indicate that this is a surface related phenomenon and not due to bulk transport.

(ii) An increased activation energy (measured at 0.04 mbar O_2_) is measured for epitaxial LSF thin films (1.23 eV) compared to a polycrystalline LSF thin film (1.00 eV), (see Fig. 6).

(iii) Similar *p*O_2_ dependencies for polycrystalline and epitaxial LSF indicate that the same mechanism dominates the oxygen exchange reaction on both structures.

(iv) For epitaxial LSF thin films, HRTEM measurements found a decrease of the out-of-plane lattice parameter with increasing LSF film thickness, while the in-plane lattice parameter stayed almost constant. For LSF grown on LSGM, the unit cell volume decreases by about 1% over the entire LSF thin film thickness.

As the oxygen exchange reaction rate on the LSF surface is heavily influenced by point defect concentrations,^22,56^ it stands to reason that the observed changes of the OER resistance are caused by a change of these defect concentrations. Based on literature, we first discuss the defect chemical changes expected from the measured decrease of the LSF unit cell volume over film thickness, *i.e.* how thermodynamic equilibrium constants are affected by lattice strain. Subsequently, we correlate the expected defect chemical changes with the observed OER resistances and activation energies.

Chemical expansion has been intensely studied in literature both on CaF_2_-type and perovskite-type electrodes.^57–66^ Whether the chemical expansion is due to size effects of vacancies or altered cation radii upon oxidation state changes is still under debate. For perovskite-type oxides, numerous studies in literature have found a strong correlation between oxygen nonstoichiometry and the crystal lattice parameters.^61,63,66–68^ A decrease of the unit cell volume is suggested to be accompanied by a decrease of the oxygen vacancy concentration in the material. However, as charge neutrality has to be maintained in LSF electrodes with constant cation stoichiometry, a change of the 
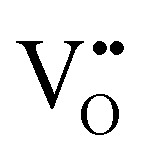
 concentration has to be counterbalanced by a change of other point defect concentrations. In general, the concentrations [i] of positively charged point defects (h˙ and 
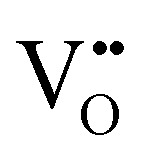
) and negatively charged point defects (e′ and 
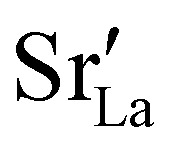
) balance each other in LSF according to1
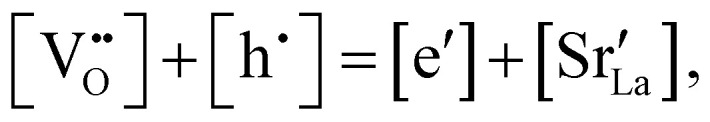
with e′ being negligible in oxidizing conditions.^56^

The equilibrium of the oxygen exchange reaction is given by2
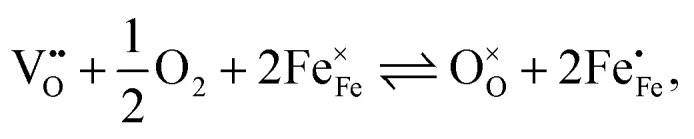
3
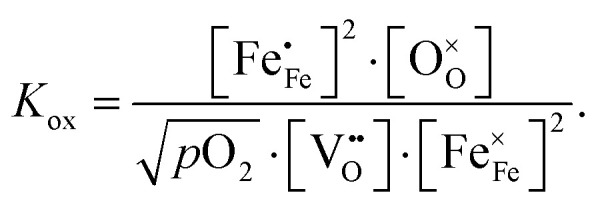


Electron holes are often considered as being localized at Fe ions or the surrounding oxygen atoms, indicated by 
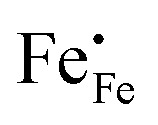
 (Fe^4+^). Moreover, the Gibbs free energy, enthalpy and entropy of oxygen incorporation (Δ*G*_ox_, Δ*H*_ox_, Δ*S*_ox_) are related to the mass action constant *K*_ox_ by:4
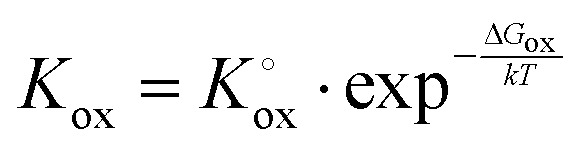

5 Δ*G*_ox_ = Δ*H*_ox_ − *T*Δ*S*_ox_

The prefactor 
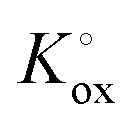
 includes the concentration of reference states. In a recent study, Lee *et al.*^30^ demonstrated the dependence of the vacancy formation enthalpy and thus of Δ*H*_ox_ on the strain state of the electrode. As stated in the equation above, an increase of *K*_ox_ (= decrease of Δ*H*_ox_) triggered by a decrease of the LSF unit cell volume leads to a lowered oxygen vacancy concentration. From eqn (1) and (3), it is further evident that a change of the oxygen vacancy concentration is accompanied by a change of the electron hole concentration.

These alterations unavoidably influence the oxygen exchange kinetics. In literature,^8,56,69^ the following equation was suggested to quantify the different contributions influencing the rates of the OER in forward or backwards reaction:6
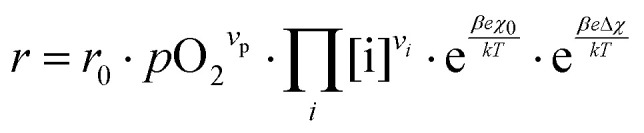


The reaction rate *r* includes the concentrations [i] of the reacting defect species *i* (with a mechanism dependent exponent *ν*_*i*_, describing the number of participating defects), the effect of oxygen partial pressure *via* adsorbates *p*O_2_ (again with a mechanism dependent exponent *ν*_p_, distinguishing atomic or molecular adsorption), the effect of any surface potential *χ*_0_ (which can become relevant if charge is transferred across the surface in this mechanism, represented by a mechanism dependent factor *β*) or its change upon an applied voltage Δ*χ*, and lastly a prefactor *r*_0_ which includes kinetic contributions (chemical activation barriers), but also thermodynamic contributions due to mass action equilibria before the rate limiting step.

In our specific case, we consider the oxygen reduction reaction direction. We assume that the surface potential *χ*_0_ does not vary with *p*O_2_ and lattice volume and that it can be included into *r*_0_ (now 
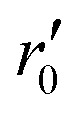
). Since no bias voltage was applied we have Δ*χ* = 0. Hence, we obtain 
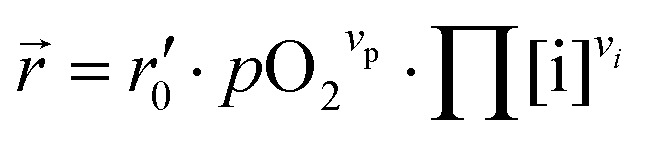
. Based on a detailed study on *p*O_2_ and voltage dependent OER resistances, a reaction mechanism was recently proposed for oxygen incorporation into LSF thin films.^22^ This includes fast molecular oxygen adsorption and first ionization of the adsorbed molecule, followed by a second ionization and dissociation as the rate limiting step. In this case, the rate law for the rate of oxygen reduction in eqn (6) can be specified as:7
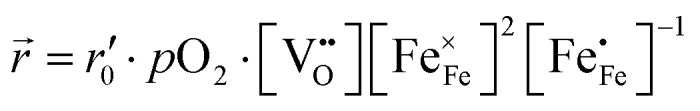


From eqn (7) it is evident that a decrease of the oxygen vacancy concentration, together with an increase of electron holes, leads to a decrease in *r⃑*, which is equivalent to an increase of the OER resistance.

**Fig. 10:**
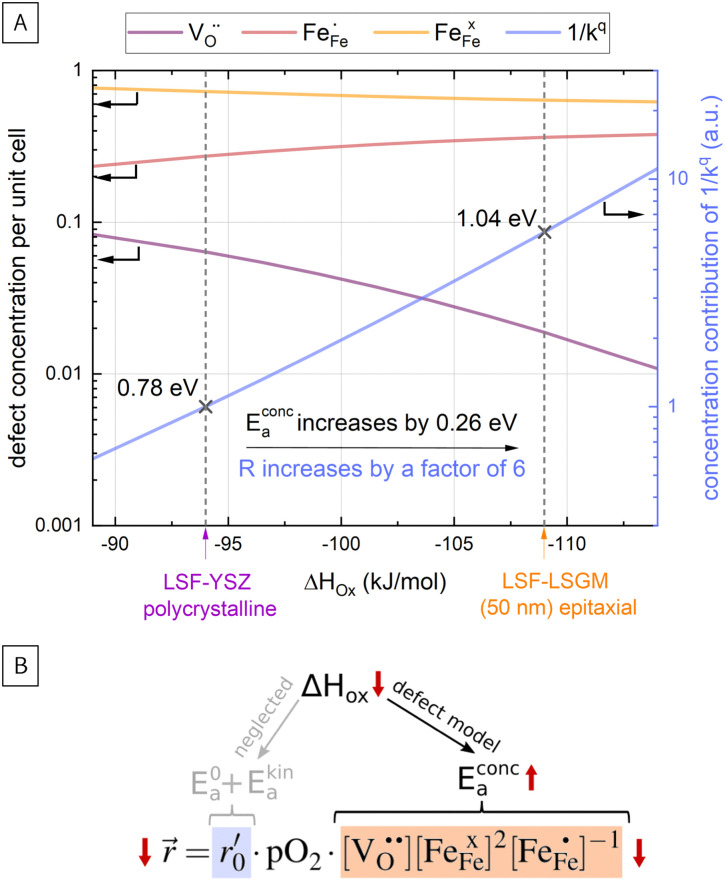
(A) Calculation of defect concentrations of LSF for different values of Δ*H*_ox_; calculation of the concentration related activation energy (*E*^conc.^_a_) and simulation of the increase of the concentration contribution to the ORR resistance (proportional to the inverse to the surface exchange coefficient), (B) sketch explaining the conducted calculations.

In Fig. 10(A), the equilibrium concentrations of 
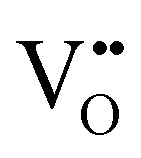
, 
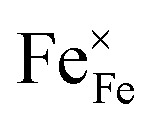
 and 
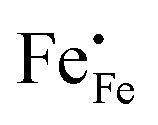
 in LSF64 electrodes are simulated for different values of Δ*H*_ox_ with Δ*S*_ox_ taken from literature.^52^ According to literature, Δ*H*_ox_ of a polycrystalline LSF thin film amounts to −94 kJ mol^−1^.^52^ The predicted concentrations for other Δ*H*_ox_ values from Fig. 10 can now be used in eqn (7) in order to estimate how much the reaction rate changes when modifying Δ*H*_ox_. The sketch in Fig. 10(B) illustrates the changes which we consider or neglect in the simulations. For the sake of simplicity, we neglect any effects of Δ*H*_ox_ on mass action constants and the kinetic barrier. Then we can determine the change of the inverse reaction rate, *i.e.* the relative change of the OER resistance in dependence of Δ*H*_ox_. The corresponding trend of the concentration contribution to the OER resistance is also shown in Fig. 10 (in arbitrary units, indicating changes but not absolute values). In our experiments we find a OER resistance increase in the range of 3–6 (see Fig. 5). According to the plot in Fig. 10(A), a decrease of Δ*H*_ox_ by 15 kJ mol^−1^ to −109 kJ mol^−1^ decreases the concentration term in eqn (7) (
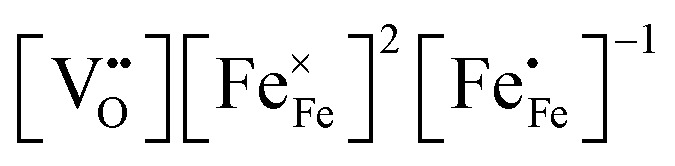
) by a factor of 6 and thus increases the ASR by a factor of six. In other words: a decrease of the oxygen incorporation enthalpy by 15 kJ mol^−1^ can explain the measured resistance change.

Defect concentrations resulting from this change of Δ*H*_ox_ were also calculated for different temperatures, yielding information on the expected changes of the ASR activation energy. The effective activation energy of the ASR itself is a non-trivial convolution of temperature dependences of all concentrations and of the thermodynamic and kinetic prefactors in the rate equation. A more detailed discussion is beyond the scope of the this paper and can be found in literature.^22^ However, we can estimate how the temperature dependency of the concentration contribution to the OER resistance changes for the adapted Δ*H*_ox_ value. This analysis shows that the activation energy contribution of the concentrations in eqn (7) increases from 0.78 eV to 1.04 eV, *i.e.* by 0.26 eV when decreasing Δ*H*_ox_ by 15 kJ mol^−1^, being very close to our measured change of 0.23 eV and further supporting our assumption that reaction rates change primarily *via* defect concentration changes. Defect simulations of an altered oxidation enthalpy are thus able to explain the results of our i-PLD measurement and also agree with the observations from HRTEM measurements, where a decrease of the LSF unit cell volume was found over film thickness.

The last issue that needs to be discussed is the driving force of the observed lattice contraction which is yet unknown. We can only speculate that a small amount of dislocations might play a role in this structural change which has not been found by HRTEM and which affect the out-of-plane lattice parameter. Also, slight cation stoichiometry changes, which have gone unnoticed in EDX measurements, might contribute to the observed changes of the out-of-plane lattice parameter. Still, as a lattice volume change usually goes hand in hand with an altered oxygen vacancy concentration, it is difficult to discuss the causality of these phenomena and the initial driving force remains unclear.

Summarizing the discussion, we presented a defect chemical model to explain the effect of lattice strain on the electrode defect concentrations and the resulting changes of the oxygen exchange kinetics and its activation energy. i-PLD measurements allowed us to precisely elucidate the effect of lattice strain on the oxygen exchange kinetics and showed that complicated structural changes can occur during epitaxial thin film growth, which have a substantial effect on the electrochemical properties of the thin film surface. It clearly shows that controlling the strain state is a valuable way for tuning the electrochemical material properties, but also emphasizes that the growth of epitaxial thin films requires a great amount of care to ensure the desired properties in the final thin film.

## Conclusion

The OER resistances of epitaxial LSF thin films on LSGM or YSZ–GDC and of polycrystalline LSF thin film electrodes on YSZ were measured during growth by *in situ* PLD impedance measurements. Results reveal that for epitaxial thin films, the OER resistance strongly increases with film thickness, up to a factor of six between 5 nm and 50 nm. The OER resistance of polycrystalline LSF electrodes, on the other hand, is hardly film thickness dependent. In addition, the activation energy of the OER resistance is significantly increased in epitaxial films. HRTEM and XRD measurements reveal a decrease of the out-of-plane lattice parameter with film thickness in the epitaxial films, which leads to a volume contraction of about 1% in 50 nm. Defect thermodynamic calculations suggest that the volume contraction of the unit cell leads to a decrease of the oxygen vacancy concentration with increasing film thickness, which subsequently causes an increased OER resistance. These defect chemical changes also are reflected in an increase of the activation energy which is in excellent agreement with experimental results. This study thus illustrates the potentially severe effect of lattice strain on the electrochemical properties of thin films, emphasizing the importance of strain for the development of highly optimized electrode and electrolyte materials in solid oxide cells and draws attention to the complexity of epitaxial thin film growth and the fact that epitaxy alone does not warrant reproducible electrochemical properties.

## Conflicts of interest

There are no conflicts of interest to declare.

## Supplementary Material

CP-025-D2CP04977E-s001
