# Supplementary material for: In situ electrochemical observation of anisotropic lattice contraction of La0.6Sr0.4FeO3−δ electrodes during pulsed laser deposition
Source: Phys Chem Chem Phys. 2022 Dec 1;25(1):142–53. doi: 10.1039/d2cp04977e (PMC9768847; doi:10.1039/d2cp04977e)
Supplement: CP-025-D2CP04977E-s001 [file CP-025-D2CP04977E-s001.pdf]

Electronic Supplementary Material (ESI) for Physical Chemistry Chemical Physics.
This journal is © the Owner Societies 2022

Supporting Information: *In-situ* electrochemical observation of anisotropic lattice contraction of $La_{0.6}Sr_{0.4}FeO_{3-\delta}$ electrodes during pulsed laser deposition

Christoph Riedl,[*a] *Matthaeus Siebenhofer,*[*‡a,c] *Sergej Raznjevic*[b], *Andreas Ewald Bumberger*[b], *Zaoli Zhang*[b], *Andreas Limbeck,*[a] *Alexander Karl Opitz,*[a] *Juergen Fleig,*[a] *Markus Kubicek*[a]

[a] Institute of Chemical Technologies and Analytics

TU Wien

Getreidemarkt 9-E164, 1060 Vienna, Austria

‡ E-mail: matthaeus.siebenhofer@tuwien.ac.at

[b] Erich Schmid Institute of Materials Science of the Austrian Academy of Sciences

Jahnstrasse 12, 8700 Leoben, Austria

[c] CEST Kompetenzzentrum für elektrochemische Oberflächentechnologie GmbH

TFZ – Wiener Neustadt Viktor-Kaplan-Strasse 2

2700 Wiener Neustadt, Austria

*Authors contributed equally.

Keywords: perovskites, MIEC, surface exchange reaction kinetics, SOFC, SOEC, electrochemical impedance spectroscopy, high resolution transmission electron microscopy, strain

1 *i*-PLD measurements of a 100 nm LSF thin film on YSZ-GDC

In the Figure 1 the OER resistance of a LSF electrode grown on YSZ-GDC is shown. It can be seen that the OER resistance decreases after reaching an electrode thickness of about 60 nm.

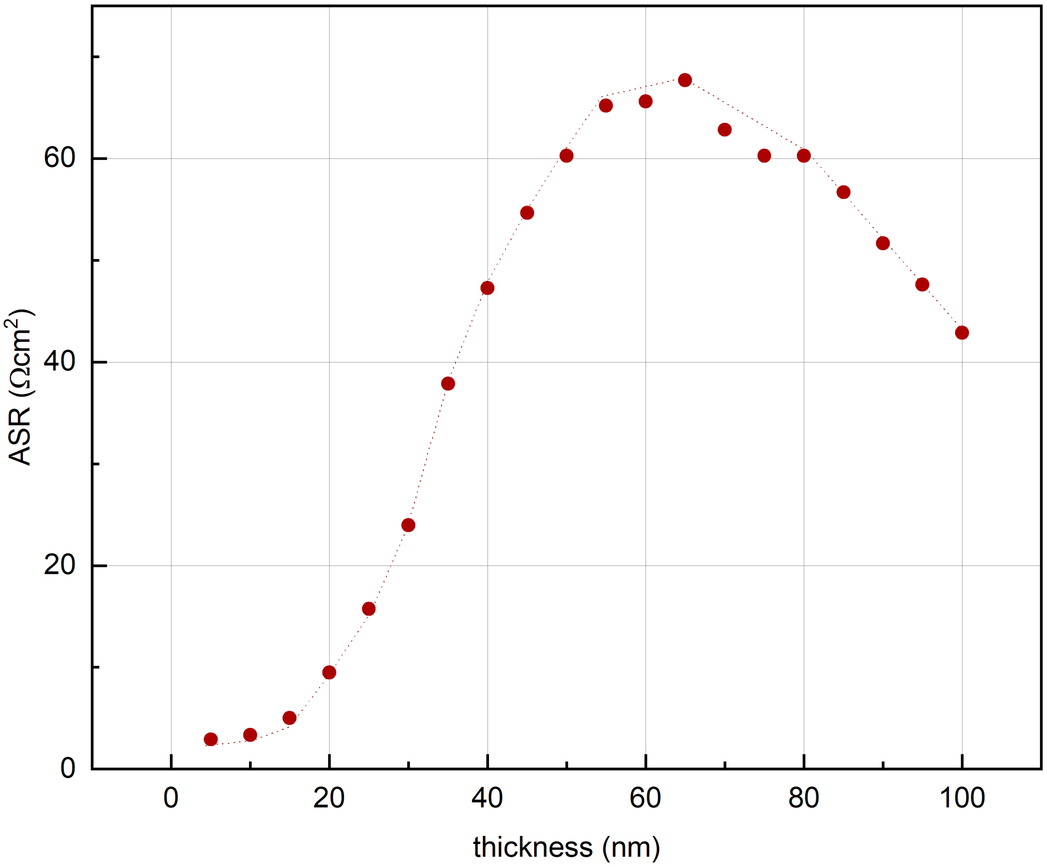

Figure 1: Area-specific resistance of a dense LSF thin film electrode grown on YSZ/GDC. Straight lines are a guide to the eye.

2 Oxygen partial pressure dependencies of epitaxial and polycrystalline LSF thin films

The oxygen partial pressure dependency of the surface exchange resistance of epitaxial and polycrystalline LSF thin films was investigated with i-PLD.

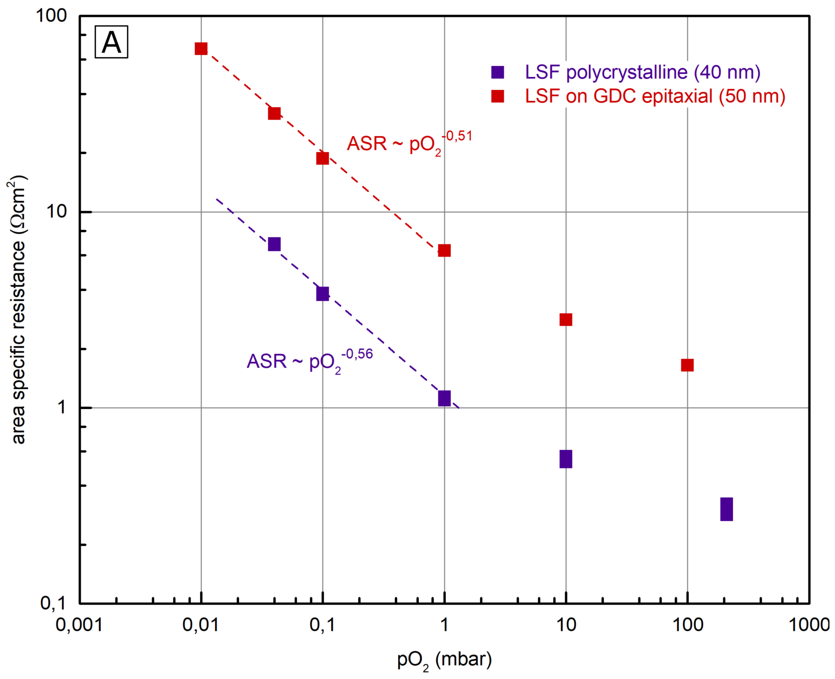

Figure 2: Oxygen partial pressure dependency of the surface exchange resistance of LSF grown on YSZ-GDC vs. LSF grown on YSZ.

3 Brownmillerite type LSF

In Figure 3, a FFT analysis of the LSF thin film is shown. Minor amounts of the brownmillerite phase were found in the entire thin film.

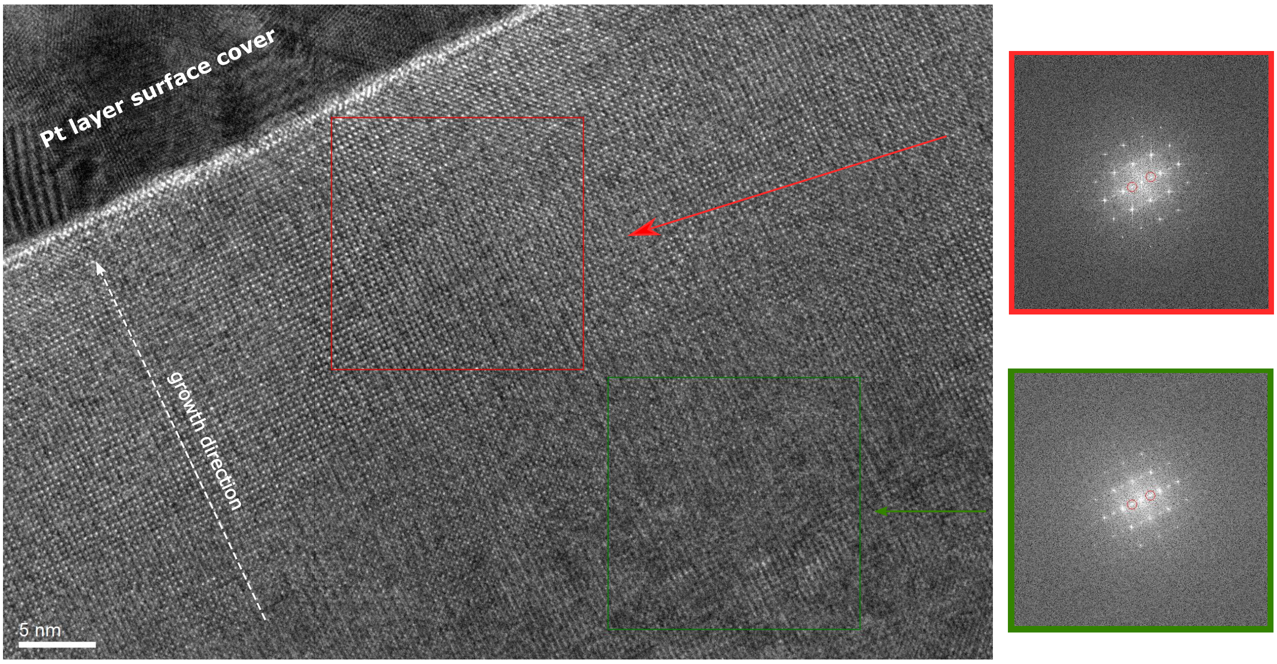

Figure 3: FFT analysis of LSF thin film. Red circles in the FFT patterns highlights the half-integer reflections indicating the presence of the brownmillerite phase.

4 Rietveld analysis

In Figure 4 a XRD diffractogram of a 50 nm thick LSF thin film grown on YSZ is shown. A Rietveld analysis was conducted to extract the lattice parameter of the polycrystalline LSF thin film.

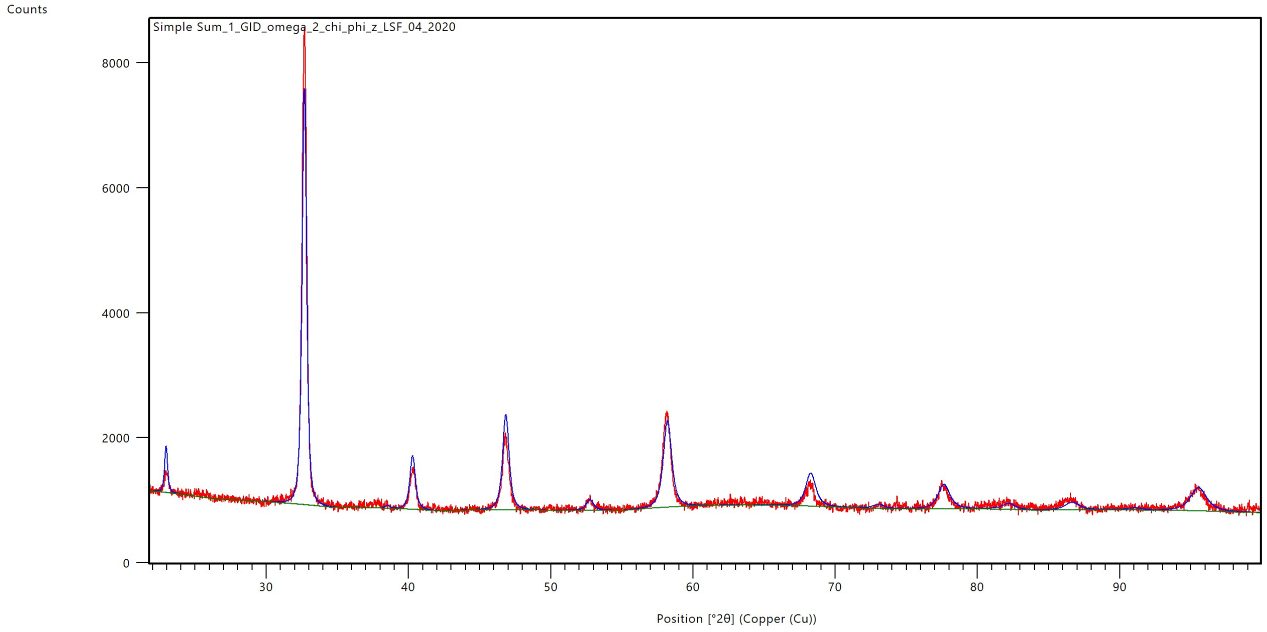

Figure 4: Rietveld analysis of LSF thin film grown on YSZ. Red line: XRD recorded in GID geometry, Blue line: Rietveld refinement Green line: background
